# A nanocomplex of C_60_ fullerene with cisplatin: design, characterization and toxicity

**DOI:** 10.3762/bjnano.8.149

**Published:** 2017-07-20

**Authors:** Svitlana Prylutska, Svitlana Politenkova, Kateryna Afanasieva, Volodymyr Korolovych, Kateryna Bogutska, Andriy Sivolob, Larysa Skivka, Maxim Evstigneev, Viktor Kostjukov, Yuriy Prylutskyy, Uwe Ritter

**Affiliations:** 1 Taras Shevchenko National University of Kyiv, Volodymyrska Str., 64, 01601 Kyiv, Ukraine; 2 School of Materials Science and Engineering, Georgia Institute of Technology, Atlanta, USA; 3 Belgorod State University, Pobedy Str. 85, 308015 Belgorod, Russia; 4 Department of Physics, Sevastopol State University, Sevastopol 299053, Crimea; 5 Technical University of Ilmenau, Institute of Chemistry and Biotechnology, Weimarer Str., 25, 98693 Ilmenau, Germany

**Keywords:** atomic force microscopy, C_60_ fullerene, cisplatin, comet assay, computer simulation, dynamic light scattering, flow cytometry, human lymphocytes, toxicity in vitro

## Abstract

The self-organization of C_60_ fullerene and cisplatin in aqueous solution was investigated using the computer simulation, dynamic light scattering and atomic force microscopy techniques. The results evidence the complexation between the two compounds. The genotoxicity of С_60_ fullerene, Cis and their complex was evaluated in vitro with the comet assay using human resting lymphocytes and lymphocytes after blast transformation. The cytotoxicity of the mentioned compounds was estimated by Annexin V/PI double staining followed by flow cytometry. The results clearly demonstrate that water-soluble C_60_ fullerene nanoparticles (0.1 mg/mL) do not induce DNA strand breaks in normal and transformed cells. C_60_ fullerene in the mixture with Cis does not influence genotoxic Cis activity in vitro, affects the cell-death mode in treated resting human lymphocytes and reduces the fraction of necrotic cells.

## Introduction

The water-soluble inorganic bi-valent platinum derivative, cisplatin (*cis*-[Pt(II)(NH_3_)_2_Cl_2_], Cis), is currently one of the most effective therapeutic agents used against cancer deceases, in particular, ovarian cancer, bladder cancer, esophagus cancer, lung cancer, and cancer of head and neck [1]. As an antitumor metal-containing agent Cis exerts an alkylating action and binds covalently to DNA. In tumor cells Cis induces the selective inhibition of DNA synthesis and replication [2]. However, the action of Cis is accompanied by side effects that limit the use of Cis in anticancer chemotherapy. Сіs-induced nephro-, hepato- and cardiotoxicity, as well as disorders of the central nervous system and sensory organs were reported [1]. Hence, there is a search for new drugs including nanodimensional compounds that could lower the side effects of Cis action, deliver Cis to the region of pathological process in a targeted manner, manage the curing at cell level, increase solubility in bioavailable form and protect Cis from degradation [3–9]. The carbon allotrope С_60_ fullerene could act as such a potent agent.

Pristine C_60_ fullerenes have no acute or sub-acute toxicity in vitro [10–12] and in vivo [13] (at least at low physiological concentrations), exerting strong antioxidant properties due to their high activity as free radical acceptors [14–15]. Water-soluble pristine С_60_ fullerenes penetrate through plasma membranes and are located in the central part of tumor cells [16]. Thereby, C_60_ fullerenes can be used for treatment of cancer [17–18], including combination chemotherapy [19] and photodynamic therapy [20–22]. They are also applied for the targeted delivery of drugs into tumor cells [23–25].

However, there are several conflicting reports in the literature regarding the genotoxicity of C_60_ fullerene [26]. Thus, a strong correlation between the genotoxic response and the concentration of an aqueous suspension of nC_60_ (178 nm in size) was observed at 2.2 µg/L in human lymphocytes using a single-cell gel electrophoresis assay [27]. In contrast, with stable C_60_ fullerene suspensions in 0.1% carboxymethylcellulose sodium or 0.1% Tween 80 aqueous solution no positive mutagenic response was observed up to the dose of 1 mg/plate with any tester strain in the bacterial genotoxicity tests in vitro and in vivo [28].

The aqueous suspension of C_60_ fullerenes caused positive responses in two bacterial genotoxicity tests, namely the Bacillus subtilis Rec-assay and umu test, up to concentrations of 0.048 mg/L and 0.43 mg/L, respectively. In [29], bulky DNA adducts could not be found by ^32^P-postlabeling/polyacrylamide gel electrophoresis assay, suggesting that an aqueous suspension of C_60_ fullerenes has the potential to damage DNA. By use of a comet assay it was also demonstrated that an aqueous suspension of C_60_ fullerenes (0.1–1 mg/L) causes a concentration-dependent increase in DNA strand breaks in haemocytes [30].

The in vivo genotoxicity of C_60_ fullerene was estimated with a comet assay in lung cells of rats. After a single and repeated instillation inflammatory responses were observed in the lungs, suggesting that C_60_ fullerene has no potential for DNA damage even at inflammation causing doses [31]. Thus, it may be concluded that the genotoxicity of C_60_ fullerene in vitro and in vivo systems may strongly depend on its concentration in biomedium, dose administration, type of cells and time of exposure.

Since the biological action of C_60_ fullerene significantly differs from the action of traditional drugs by the mechanism of penetration inside cells and biodistribution [23–25,32–35], the conjugation of С_60_ molecules with drugs is currently considered a perspective biomedical strategy. The formation of a stable non-covalent nanocomplex of С_60_ fullerene with doxorubicin (C_60_+Dox) in aqueous solution was confirmed theoretically and experimentally [23,34,36]. The antitumor action of the C_60_+Dox nanocomplex was reported to be stronger than the sole action of Dox or С_60_ fullerene in vivo [23–24]. Moreover, recently it was found that C_60_ fullerene in C_60_+Dox nanocomplex prevents cyto- and genotoxic effects of Dox on lymphocytes in vitro [37–38]. Based on these results it was suggested that the mechanism of complexation could induce biological synergy for other drugs administered together with C_60_ fullerene as well [19,23]. Taking into account the importance of Cis in chemotherapy of cancer, this drug could be a candidate molecule for study. A recent extended physico-chemical study has confirmed the formation of non-covalent entropically driven nanocomplexes between Cis and C_60_ fullerene in physiological solution (i.e., the adsorption of Cis in C_60_ fullerene clusters) [25,39]. Hence, it is reasonable to expect the biological interaction of these drugs. In order to testify this hypothesis in the present study we evaluated and compared in vitro cytotoxic action of C_60_ fullerene, Сіs and their complex on lymphocytes from healthy persons, as well as their genotoxic effects towards resting lymphocytes and lymphocytes after blast transformation.

## Experimental

### Materials preparation

A highly stable reproducible aqueous colloid solution of pristine C_60_ fullerene (C_60_FAS) with a maximum concentration of 0.15 mg/mL was prepared according to the protocol [40–41]. The initial stock solution of Cis (“Сisplatin-TEVA”, Pharmachemie B.V.) was prepared with a concentration of 0.5 mg/mL and was further diluted to the required concentrations used in particular experiments.

Immobilization of Cis on С_60_ fullerene was accomplished according to the following protocol: C_60_FAS and Cis solution were mixed in a molar ratio of 1:2.4 (typically 0.1 mM С_60_ fullerene and 0.24 mM Cis). The obtained mixture was subjected to ultrasonic treatment in dispersant for 20 min, followed by magnetic stirring over 12 h at room temperature.

### Computer simulation

The spatial structure of the C_60_ fullerene was built according to [http://www-jmg.ch.cam.ac.uk/data/molecules/misc/c60.html]. The spatial structure of Cis was built with the aid of HyperChem 8.0 according to Wysokiński et al. [42] and then optimizated in Gaussian 09W at the mPW1PW hybrid level of theory [43] in LanL2DZ basis set [44]. This level of theory and basis set is considered to be optimal for quantum-mechanical calculations of the molecules containing platinum atoms, in particular for Cis [42]. The spatial structure of the C_60_+Cis nanocomplex was built according to Kostjukov et al. [45] with the aid of the XPLOR software (version 3.851 [46] with CHARMM27 force field). The plane of the Cis molecule was located parallel to the surface of the C_60_ fullereneat a distance of ca. 3.4 Å. Geometry optimization of the C_60_+Cis nanocomplex was accomplished by means of molecular mechanics in X-PLOR. The modeling of the aqueous environment was carried out by water molecules in the form of TIP3P placed in a cubic box with a side length of 35 Å (1423 molecules).

#### DLS study

Measurement of the hydrodynamic size distribution was performed by dynamic light scattering (DLS) on a Zetasizer Nano ZS (Malvern Ins. Ltd) with upload of multiple narrow modes (high resolution) at room temperature. The instrument is equipped with a He–Ne gas laser (max. output power 5 mW) operating at a wavelength of 633 nm. The measurements were performed at a 173° scattering angle (NIBS technology). The autocorrelation function of the scattered light intensity was analyzed by the Malvern Zetasizer software.

The zeta potential was measured with a Zetasizer Nano ZS (Malvern Ins. Ltd) using a universal dip cell in disposable cuvettes. The Smoluchowski approximation was used to convert the electrophoretic mobility to the zeta potential.

#### AFM study

The surface morphology of the particles was examined using atomic force microscopy (AFM). AFM images were collected using an Integra Spectra microscope (NTMDT, Russia) in the “light” tapping mode according to the well-established procedure. For the sample preparation, a drop of solution was placed onto a pre-cleaned microscope glass slide and dried in air prior to AFM imaging.

### Cell isolation and cultivation

Human peripheral blood from healthy donors was collected into a heparinized medical syringe. Lymphocytes were separated by centrifugation in a density gradient (Histopaque 1077, Sigma, USA) according to instructions of the manufacturer and washed twice: control lymphocytes in 0.15 M NaCl, lymphocytes that were intended for blast transformation reaction in RPMI 1640 medium. To induce the blast transformation the lymphocyte suspension was cultivated in RPMI 1640 medium with 10% FBS and 1000 units/mL IL-2α at 37 °C for 20 h. After cultivation the cells were washed in 0.15 M NaCl. Aliquots of the suspension were used for cytological analysis to evaluate the level of blast transformation (the fraction of lymphoblasts).

#### Incubation of lymphocytes and lymphoblasts

The cell suspension in RPMI 1640 medium (cell concentration in the range of 1 × 10^5^ to 5 × 10^5^ cells per mL) was incubated in the presence of either C_60_ fullerene (0.1 mg/mL), anticancer drug Сіs (0.01, 0.1 or 0.15 mg/mL) or the complex of C_60_ fullerene with Cis (Cis concentration was 0.1 or 0.15 mg/L, the C_60_ fullerene to Cis molar ratio was equal to 1:2.4) for 1.5 h at 37 °C, washed once in 0.15 M NaCl, and then used for the comet assay. Five to seven independent repeats of the experiments were performed. As shown before [25], the molar ratio of 1:2.4 yields the highest anticancer activity of the С_60_+Cis complex and was therefore used in the experiments.

#### Comet assay

To obtain lysed cells (nucleoids) 20 µL of the cell suspension was mixed with 40 µL of 1% low-melting agarose (Sigma, USA) at ca. 37 °C. 20 µL of the mixture were used to prepare a microscope slide previously covered with 1% high-melting agarose. After agarose polymerization, the slides were placed in the lysis solution consisting of 2.5 М NaCl, 100 mM ЕDTA, 10 mM Tris-HCl (рН 7.5), and 1% Triton X-100 (Ferak, Germany), which was added before use. Cells were exposed to lysis solution for 2 h at 4 °C. After the lysis, slides were washed with TBE buffer (89 mM Tris-borate, 2 mM EDTA, рН 7.5) and electrophoresed in the same buffer for 20 min at 4 °C (1 V/cm, 300 mA).

After electrophoresis, the slides were stained with 1.3 μg/mL of DAPI (Sigma, USA) and immediately analyzed under a fluorescence microscope (LOMO, Russia) connected with Canon A570 camera (a total 200 to 300 cells on each slide were analyzed). The relative amount of DNA in the comet tail, the parameter that reflects the level of DNA damages, was determined using the image analysis software programs Comet Assay IV (Perspective Instruments, UK) and CometScore (TriTec Corp., USA).

#### Cell-death assay

Apoptosis was assessed by staining cells with Annexin V–fluorescein isothiocyanate (FITC) and counterstaining with propidium iodide (PI) with the use Annexin V-FITC Apoptosis Detection Kit (Dojindo EU GmbH, Munich, Germany) according to the instructions of the manufacturer. Briefly, 2 × 10^5^ cells were placed into wells of a 96-well flat-bottom plate and were treated with C_60_ fullerene (sample 1), Сіs (sample 2) and C_60_+Cis nanocomplex (sample 3) for 24 h. All additives were used at the concentration of 0.15 mg/mL. Untreated cells were used as a control (sample 4). Afterwards cells were washed twice with PBS and incubated in the Annexin V binding buffer containing 1/50 volume of FITC-conjugated Annexin V solution and PI (50 µg/mL) for 10 min at room temperature in the dark. Cells from each sample were then analyzed by FacsCalibur flow cytometer (BD Biosciences). The data were analyzed using CELLQuest software (BD). PI detects cells that have lost CPM integrity (i.e., necrotic and secondary necrotic cells), whereas Annexin V detects early apoptotic cells.

### Statistics

Statistical analysis was performed by conventional methods of variation statistics. Significance of the differences between the control and experimental measurements was estimated within the framework of the Student’s t-test using Origin 8.0 software (OriginLab Corporation, USA). The difference between the compared values was considered to be significant at *p* < 0.05.

## Results and Discussion

### Characterization of the C_60_+Cis mixture

The freshly prepared mixture of C_60_ fullerene with Cis was characterized by conventional physico-chemical methods, namely DLS and AFM. The monitoring of the morphology of nanoparticles in solution is important not only for checking the quality of solution for study, but also to control the degree of aggregation which may influence their biodistribution and toxicity [47].

**Figure 1:**
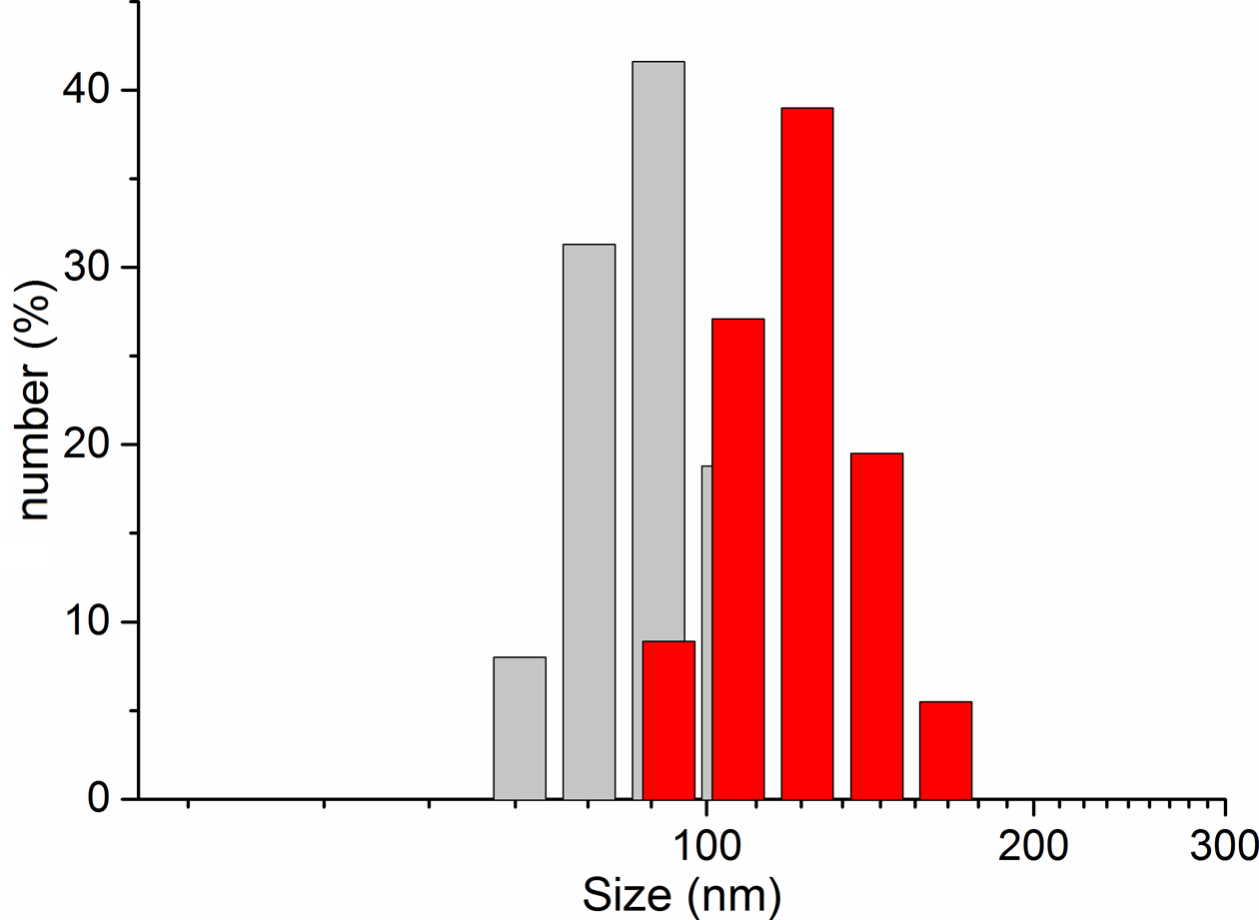
DLS (hydrodynamic size) results of C_60_FAS (grey; concentration 0.15 mg/mL) and C_60_+Cis mixture (red; molar ratio of 1:2.4).

**Figure 2:**
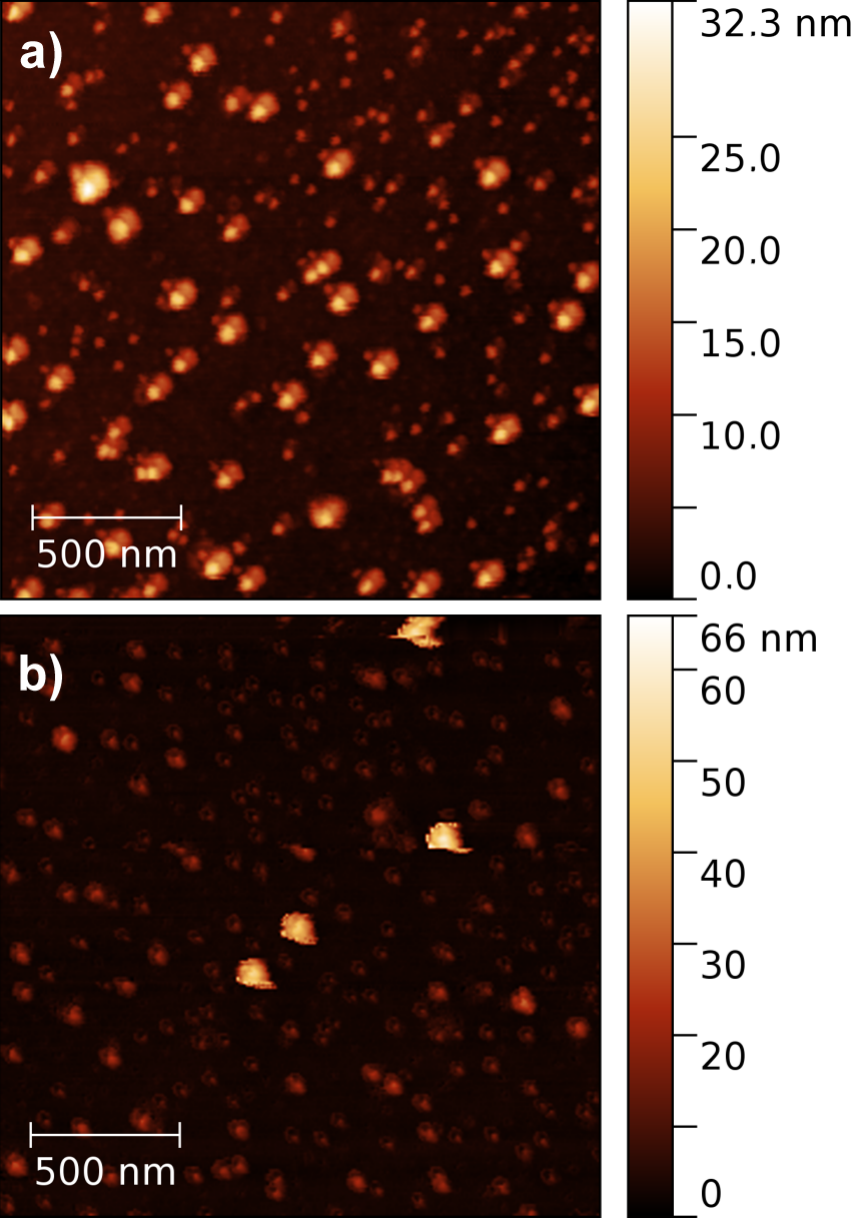
AFM images of a) nanoparticles in C_60_FAS (concentration 0.15 mg/mL) and b) C_60_+Cis mixture (molar ratio as 1:2.4).

Figure 1 shows DLS data of C_60_FAS and C_60_+Cis mixture at room temperature. It is seen that C_60_FAS contains C_60_ fullerene nanoparticles with hydrodynamic sizes ranging from 65 to 105 nm. The C_60_+Cis nanocomplex exhibits hydrodynamic sizes from 91 to 164 nm. The *Z*-average size of the C_60_+Cis nanocomplex is about 122 nm. These results are in accordance with AFM data (Figure 2), as well as with previous study of C_60_+Cis complexation [39].

The zeta potential of the C_60_+Cis mixture measured in this work equals to −16.8 mV at room temperature. It is known from previous studies that C_60_ fullerene clusters not containing any guest molecules have a zeta potential equal to −23 mV in water solution [41]. Addition of neutral Cis molecules into C_60_FAS results in their adsorption into the C_60_ fullerene clusters and causes a lowering of the absolute value of the zeta potential. The stability of such negatively charged clusters in water is determined by two opposite forces, viz., electrostatic repulsion of negatively charged C_60_ molecules and attraction of the C_60_ fullerenes due to hydrophobic and van der Waals forces. Thereby, the negative potential of C_60_+Cis clusters is an important factor responsible for the stabilization of this aqueous system.

**Figure 3:**
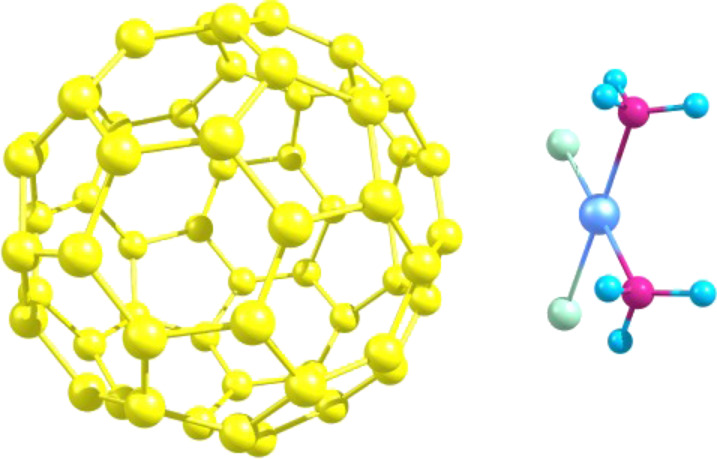
The calculated energy-optimized structure of the C_60_+Cis nanocomplex in aqueous solution.

The structural and energetic peculiarities of C_60_+Cis complexation were investigated by calculating the energy-minimized spatial structure of their complex, shown in Figure 3.

The initial location coordinates of Cis above the C_60_ fullerene surface were taken from the ab initio structure [39]. Then we performed the molecular dynamics simulation of this nanocomplex in aqueous environment and calculated the time-averaged energies of interaction. The net van der Waals, electrostatic and hydrophobic energies were obtained as follows, Δ*G*_vdw_ ≈ −0.6 kJ/mol, Δ*G*_el_ ≈ 0.9 kJ/mol and Δ*G*_hyd_ ≈ −9.0 kJ/mol, respectively. The near-zero magnitudes of the net ‘vdw’ and ‘el’ terms are quite expected and originate from compensatory nature of the enthalpic interaction with water environment and between the interacting molecules (discussed in more detail in [36,39]). The ‘hyd’ term outweighs any other interactions indicating the predominantly entropic character of C_60_+Cis complexation. The obtained results fully agree with previous calorimetric measurements of the same system [39] reporting the purely hydrophobic nature of interaction between these molecules. Moreover, the same conclusion was made regarding the aggregation of C_60_ fullerene in solution [48], C_60_ fullerene complexation with Dox [36] and landomycin A [49], and seems to reveal a general pattern of complexation of small molecules in water [45].

### Estimation of genotoxic effects

**Figure 4:**
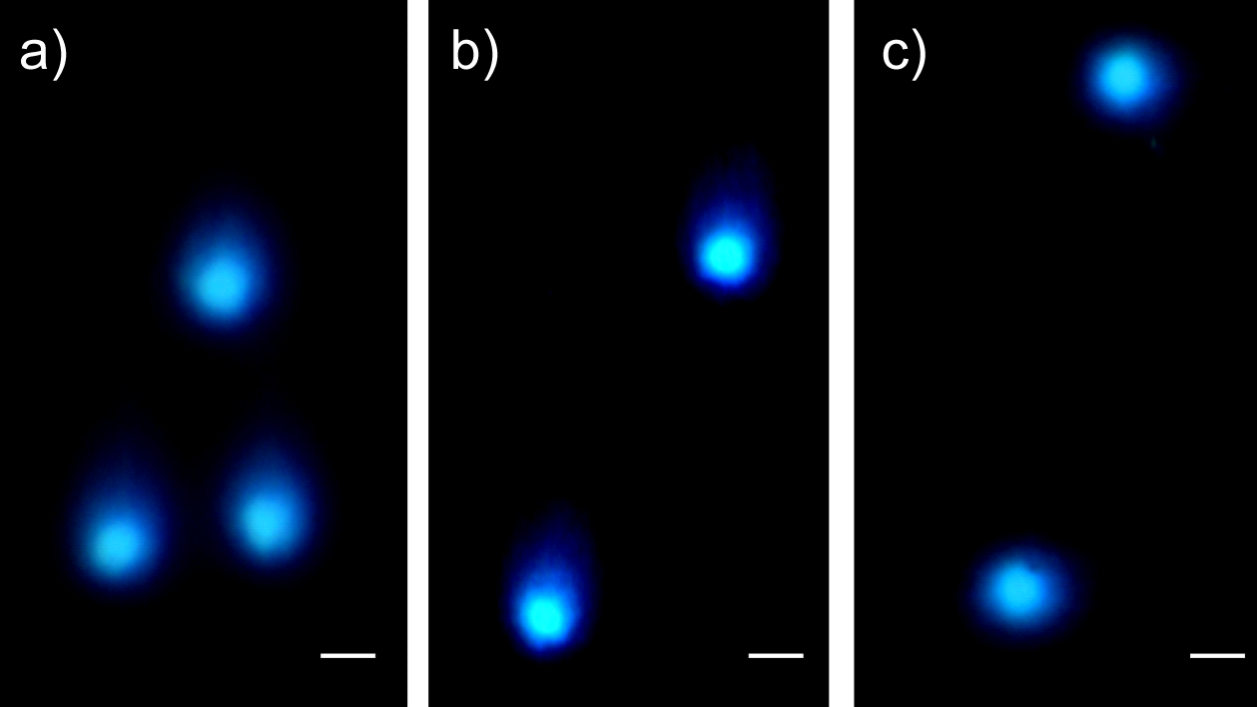
The representative comet-assay images obtained after 20 min of electrophoresis of a) control cells, b) cells incubated with C_60_ fullerene at concentration of 0.1 mg/mL, and c) cells treated with Cis at 0.15 mg/mL. The bars correspond to 10 µm.

Figure 4 shows typical images of the comet assay obtained after 20 min of electrophoresis of lysed cells. For both lymphocytes and lymphoblasts, either the control cells or cells treated with the agents studied, we did not observe any differences in the comet appearance.

**Figure 5:**
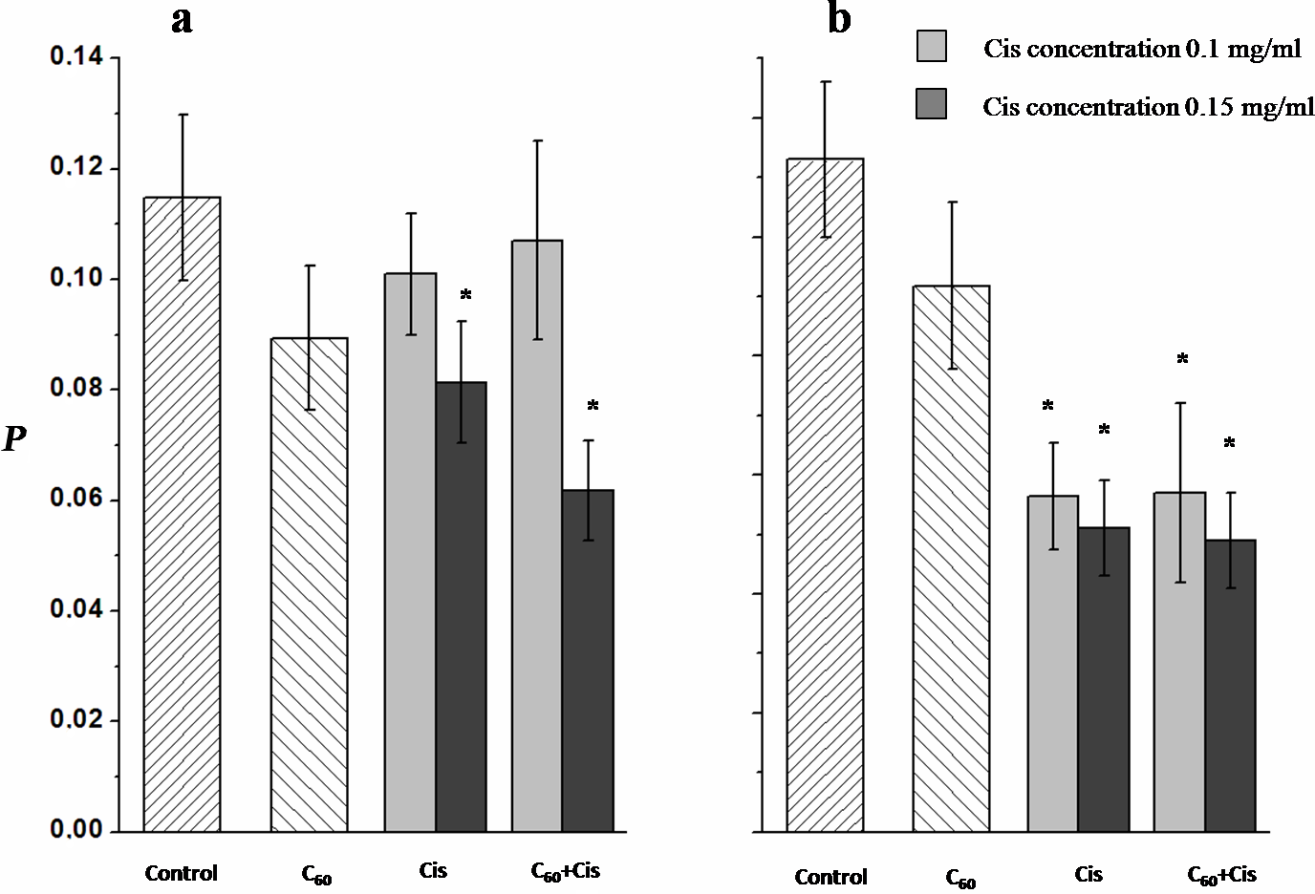
The relative amount of DNA in the comet tails (*P*) after 20 min of electrophoresis of a) lymphocytes and b) lymphoblasts treated with Cis, C_60_ fullerene or C_60_+Cis nanocomplex. Control: cells were incubated in RPMI 1640 medium without any additional agents. The average values of 5–7 independent experiments are presented. The error bars represent the standard deviations. *Statistically significant (*p* < 0.05) with respect to control cells.

The average amount of DNA in the comet tails in control experiments, when the isolated lymphocytes or lymphoblasts were incubated in RPMI 1640 medium without any agents, was ca. 0.11 for both cell types (Figure 5). This value, which appears to be slightly higher than that usually observed for intact cells (the typical value is 0.06–0.07) [50], may indicate that a small amount of DNA strand breaks occurred in the cells. We did not observe any significant changes in the average amount of DNA in the comet tails after cell treatment with C_60_ fullerene (Figure 5). Thus, C_60_ fullerene nanoparticles do not induce the DNA breaks in the cells.

At a low Cis concentration (0.01 mg/mL) the Cis-treated lymphocytes and lymphoblasts showed a DNA amount in the tails comparable to that of control cells. The same picture was observed for lymphocytes treated with Сis at 0.1 mg/mL (Figure 5a), but a significant decrease in the DNA fraction in the comet tails was detected for lymphoblasts incubated with Cis at this concentration. To explain this result it is worth remembering the mechanism of Cis action. After penetration into cell nuclei Cis may induce coordinate bonds between Pt and guanine bases in DNA that leads to intra- and inter-strand crosslinking. In addition, Cis interaction with nuclear proteins induces DNA–protein crosslinking. After cell lysis these crosslinks remain in nucleoids, which hamper DNA migration in the comet tail under electrophoretic conditions, i.e., the lower the fraction of DNA in the tail, the stronger the mutagenic action of Cis. Thus, lymphoblasts appear to be more sensitive to Cis action than lymphocytes. During cultivation with IL-2α (when lymphocytes are transformed into lymphoblast) a large set of genes are activated to allow the entry of cells in the G1 phase of the cell cycle [51]. Probably, such transformation that never occurs in vivo in lymphocytes under normal conditions, leads to an increase in the cells' sensitivity to the anticancer drug Cis.

The increase of the Cis concentration up to 0.15 mg/mL causes significant decrease in the DNA fraction in the comet tails for both cell types, viz., the average amount of DNA in the tail was 0.08 ± 0.01 for lymphocytes and 0.05 ± 0.01 for lymphoblasts. At the same time, we did not observe any differences in DNA fraction in the comet tail between cells treated with Cis only or with its nanocomplex with C_60_ fullerene. Hence, C_60_ fullerene in the nanocomplex does not influence the Cis activity.

### Comparative evaluation of the cytotoxic effects

**Figure 6:**
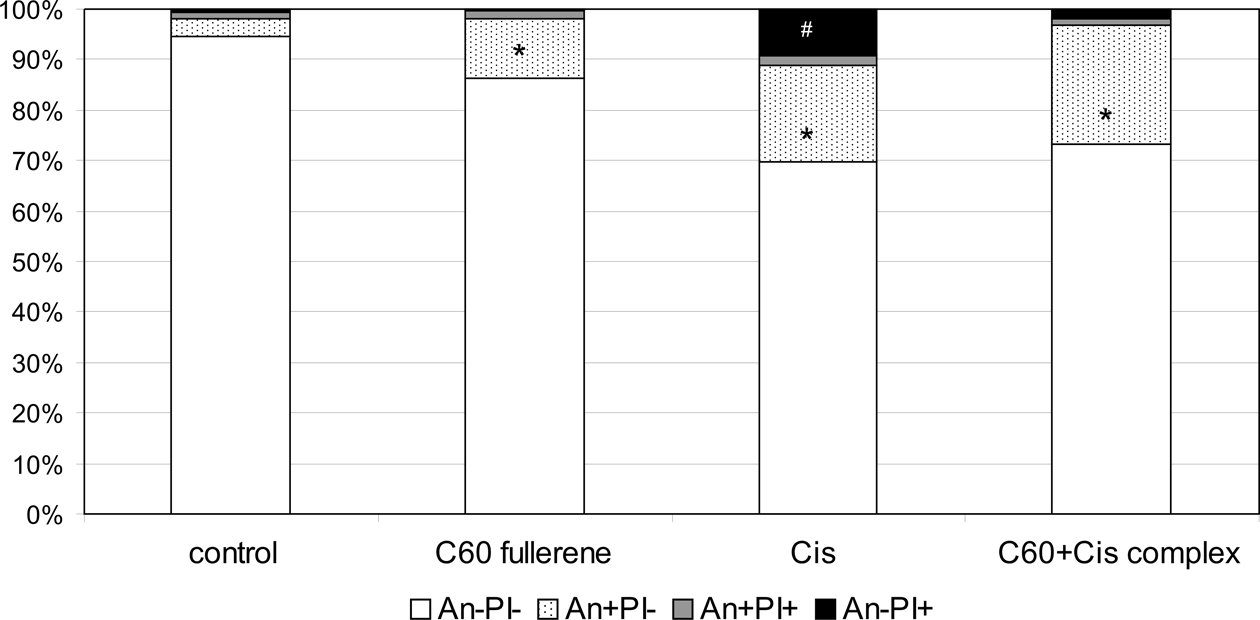
C_60_ fullerene, Cis and their nanocomplex induce apoptosis as well as necrosis of lymphocytes from healthy persons. Cells were treated with mentioned compounds at the concentration of 0.15 mg/mL for 24 h. After culturing, cells were stained with annexin V (AnnV)/propidium iodide (PI) and analyzed by flow cytometry. Control: cells were incubated without any additional agents. The average values for four independent experiments are presented. *p < 0.05 compared with untreated cells; ^#^*p* < 0.05 compared with cells treated with C_60_+Cis nanocomplex.

Genotoxic effect of Cis is mostly associated with apoptotic cell death. However, mechanism of Cis cytotoxic action involves multiple signaling pathways inducing not only apoptosis but also necrotic cell death [52–55]. Nephrotoxicity is considered to be the most important side effect of Cis and is mainly caused by tubular epithelial cell necrosis induced by extensive reactive oxygen species (ROS) generation [56–57]. According to Kaeidi et al. [58], preconditioning with mild oxidative stress may enhance some endogenous defense mechanisms and stimulate cellular adaptation to subsequent severe oxidative stress after the treatment with Cis. C_60_ fullerene can either consume ROS or induce their generation [59]. Taking into account this fact we have hypothesized that C_60_ fullerene in the nanocomplex with Cis can affect mode of cell death induced by Cis. In order to testify this hypothesis, Annexin V/PI double staining of human healthy lymphocytes treated with either C_60_ fullerene, Cis or their nanocomplex was conducted. As shown in Figure 6, the total number of dead lymphocytes from healthy persons after the treatment with C_60_ fullerene was 13.8% vs 32.4% and 36.7% in samples of cells treated with Cis and C_60_+Cis nanocomplex, respectively.

Analysis of cell death using an Annexin V-FITC/PI assay allows one to differentiate the stages of apoptosis and to reveal necrotic cells. The treatment of human healthy resting lymphocytes with C_60_ fullerene resulted in significant increase of early apoptotic cells (An+PI−) to 11.8%, and raise of late apoptotic (An+PI+) to 1.7% on average, as well as necrotic cells (An−PI+) to 0.3%. Apoptosis to necrosis ratio in these samples was 6:1 (on average). In cell samples treated with Cis we noticed significantly more necrotic cells (9.2%), wherein apoptosis to necrosis ratio was 2:1. C_60_+Cis nanocomplex induced mainly apoptosis in resting lymphocytes, and apoptosis to necrosis ratio was 7:1.

## Conclusion

1. The computer simulation, DLS and AFM data confirmed the ability of C_60_ fullerene to form non-covalent nanocomplex with Cis in aqueous solution.

2. C_60_ fullerene nanoparticles do not induce DNA strand breaks in the normal (lymphocytes) and transformed (lymphoblasts) cells as revealed by the comet assay.

3. C_60_ fullerene in the C_60_+Cis nanocomplex does not influence the genotoxic activity of Cis in vitro.

4. C_60_ fullerene in the C_60_+Cis nanocomplex affects the cell death mode in treated resting lymphocytes from healthy persons and reduces the fraction of necrotic cells.
